# Temporal trends in the prescription of low-dose antithrombotic and anti-inflammatory therapies in Germany (2022–2024)

**DOI:** 10.1007/s00392-025-02761-x

**Published:** 2025-10-07

**Authors:** Michael Kunz, Andrea Espinosa Daudí, Marita Kieble, Ulrich Laufs, Felix Götzinger, Jasper Boeddinghaus, Gregor Leibundgut, Christoph R. Meier, Christian Müller, Martin Schulz, Felix Mahfoud

**Affiliations:** 1 Department of Cardiology, University Heart Center Basel, University Hospital Basel, Petersgraben 4, 4031 Basel, Switzerland; 2 Cardiovascular Research Institute Basel (CRIB), 4056 Basel, Switzerland; 3 German Institute for Drug Use Evaluation (DAPI), 10557 Berlin, Germany; 4 Klinik Und Poliklinik Für Kardiologie, Universitätsklinikum Leipzig, 04103 Leipzig, Germany; 5 Clinical Pharmacy & Epidemiology, Department of Pharmaceutical Sciences, University of Basel, 4056 Basel, Switzerland; 6 Hospital Pharmacy, University Hospital Basel, 4031 Basel, Switzerland; 7 Institute of Pharmacy, Freie Universität Berlin, 12169 Berlin, Germany

**Keywords:** Coronary artery disease, Prolonged antithrombotic therapy, Antiplatelet therapy, Guideline-directed medical therapy, Drug utilization

## Abstract

**Aims:**

This study aimed to investigate the prescription rates of low-dose antithrombotic and anti-inflammatory therapies in Germany from 2022 to 2024.

**Methods:**

We analyzed national outpatient claims data from the German Institute for Drug Use Evaluation. Claims data for patients receiving the following medications were included: aspirin 100 mg, clopidogrel 75 mg, prasugrel 5 mg and 10 mg, ticagrelor 60 mg and 90 mg, rivaroxaban 2.5 mg, and colchicine 0.5 mg. Drug utilization was quantified using defined daily doses per 1000 Statutory Health-insured persons per day (DID, 10 DID = approximately 1% of the population receive the drug daily).

**Results:**

In 2024, aspirin had the highest dispensing volume (47.4 DID), followed by clopidogrel (10.6 DID). All other substances were infrequently prescribed: prasugrel 5 mg (0.065 DID) and 10 mg (1.388 DID), ticagrelor 60 mg (0.103 DID) and 90 mg (0.978 DID), rivaroxaban 2.5 mg (1.535 DID), and colchicine (0.457 DID). Rivaroxaban 2.5 mg was the only drug showing a notable increase in utilization over time (+ 42% from 2022 to 2024). Cardiologists accounted for a small proportion of prescriptions (mean DID share 3.3%, 1.8% of all dispensed DDD). Aspirin use increased consistently with age, while rivaroxaban 2.5 mg peaked in the 70–74 age group. In contrast, ticagrelor 60 mg was more frequently prescribed in middle-aged patients.

**Conclusion:**

Despite the high prevalence of coronary artery disease (CAD), the use of long-term antithrombotic and anti-inflammatory therapies for secondary prevention in patients with CAD remains low in Germany, especially among older patients.

**Graphical abstract:**

Temporal trends in the prescription of low dose antithrombotic and anti-inflammatory therapies in Germany (2022–2024). ASA = aspirin, CAD = coronary artery disease, DAPI = German Institute for Drug Use Evaluation (Deutsches Arzneiprüfungsinstitut e.V.), DDD = Defined Daily Dose, DID = Defined Daily Dose per 1000 inhabitants per day, ESC = European society of cardiology, SHI = statutory health insurance

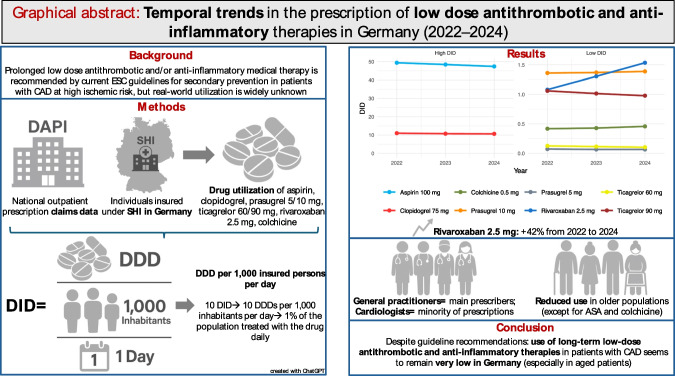

**Supplementary Information:**

The online version contains supplementary material available at 10.1007/s00392-025-02761-x.

## Introduction

Despite advances in revascularization techniques and secondary prevention strategies, patients with chronic coronary syndrome (CCS) and/or a history of acute coronary syndrome (ACS) continue to face a substantial residual risk of cardiovascular events [1]. However, the use or non-use of evidence-based advanced pharmacotherapy is not fully understood.

In addition to established risk factors, chronic low-grade inflammation has emerged as a key contributor to the progression of atherosclerosis [2]. The intensification of antithrombotic and/or anti-inflammatory strategies can reduce ischemic risk beyond standard care [3–7]. The combination of low-dose rivaroxaban and aspirin significantly reduced the risk of cardiovascular death, stroke, or myocardial infarction (MI) in patients with stable atherosclerotic cardiovascular disease (ASCVD) compared to aspirin alone, albeit with an increased risk of major bleeding [6]. Similarly, the long-term use of ticagrelor in addition to aspirin in patients with a prior MI reduced ischemic events, with a manageable bleeding risk [5]. In addition, a prasugrel-based de-escalation strategy was non-inferior in preventing ischemic events but significantly reduced bleeding, supporting the role of low-dose prasugrel in the maintenance phase of secondary prevention after ACS [8]. Extended dual antiplatelet therapy (DAPT) to 30 months after drug-eluting stent (DES) implantation significantly reduced stent thrombosis and ischemic events [7]. Evidence supporting the use of prasugrel 5 mg as long-term maintenance therapy after ACS is also provided by the 4D-ACS trial, in which prasugrel 5 mg monotherapy after one month of DAPT was non-inferior and superior to continued DAPT for the prevention of ischemic events and resulted in fewer bleeding complications [9]. These findings have led to guideline recommendations for extended dual antithrombotic therapy (DAT) in selected high-risk patients [10, 11]. Beyond antithrombotic strategies, low-dose colchicine is recommended by the current guidelines of the European Society of Cardiology (ESC) for patients with atherosclerotic CAD to reduce myocardial infarction, stroke, and the need for revascularization with a class IIa-A recommendation [4, 11, 12].

Despite these findings and corresponding recommendations in the ESC guidelines [10–12], the adoption of these strategies, such as low-dose rivaroxaban (2.5 mg twice daily), ticagrelor 60 mg, or prasugrel 5 mg beyond 12 months post-ACS, and 0.5 mg colchicine daily, appears infrequently prescribed in patients with established CAD and high ischemic risk. However, this impression is largely anecdotal and not supported by comprehensive prescription data. To address this gap, we analyzed claims data to investigate real-world utilization of aspirin, clopidogrel, ticagrelor, prasugrel, low-dose rivaroxaban, and colchicine from 2022 to 2024 in Germany (Graphical abstract).

## Methods

### Study design and inclusion criteria

This analysis used data from the German Institute for Drug Use Evaluation (Deutsches Arzneiprüfungsinstitut e.V., DAPI, www.dapi.de) from 2022 to 2024. The database contains anonymized dispensing data from more than 95% of Germany’s community pharmacies, claimed at the expense of the Statutory Health (SH) Insurance funds. The available data were extrapolated by regional factors to represent all SH-insured persons—that is, approximately 87% of the total German population. The database contains outpatient data only, and prescriptions for privately insured patients are not covered. Except for clustered years of birth, no data on individual patients (e.g., sex) were available. Only prescriptions dispensed to individuals aged 45 years or older were considered to focus on age groups with a higher risk for CAD.

### Consent

This study was conducted in accordance with the ethical principles outlined in the Declaration of Helsinki. As no individual patient-level information was accessed, ethical approval and informed consent were not required.

### Included drugs and statistical methods

Included in the analyses were aspirin (acetylsalicylic acid) 100 mg, ticagrelor 60 and 90 mg, prasugrel 5 and 10 mg, rivaroxaban 2.5 mg, clopidogrel 75 mg, and colchicine 0.5 mg (Table 1). Only pharmaceutical products for oral administration in solid dosage forms were included. Anatomical Therapeutic Chemical (ATC) codes were used to define and select relevant monotherapies for inclusion. Based on the evidence from the DAPT trial [7] and the 4D-ACS trial [9], prasugrel 5 mg was included among the investigated medications. The current ESC guidelines for CCS recommend a reduced dose of prasugrel (5 mg once daily) in patients with a body weight < 60 kg or age ≥ 75 years [12].

**Table 1:** Included drugs, analyzed doses per solid oral dosage forms and applied defined daily doses (DDD)

Drug	ATC code	Dose	Defined daily dose
Aspirin (acetylsalicylic acid)	B01AC06, N02BA01	100 mg	100 mg
Ticagrelor	B01AC24	60 mg	120 mg*
Ticagrelor	B01AC24	90 mg	180 mg
Prasugrel	B01AC22	5 mg	5 mg*
Prasugrel	B01AC22	10 mg	10 mg
Clopidogrel	B01AC04	75 mg	75 mg
Rivaroxaban	B01AF01	2.5 mg	5 mg*
Colchicine	M04AC01	0.5 mg	0.5 mg*

^*^DDD defined for this study

*ATC* Anatomical Therapeutic Chemical (ATC) classification system

Drug utilization was expressed as defined daily doses (DDD) per 1000 SH-insured persons per day (DID = DDD/1000 inhabitants/day) providing a standardized measure of drug exposure over time, following established methodology [13, 14]. The number of individuals insured under the SHI was obtained from the German Federal Ministry of Health. According to the WHO, the DID is particularly suitable for medications used in chronic conditions, assuming a close match between the prescribed daily dose and the DDD. For illustration, a value of 10 DID corresponds to 10 DDDs per 1000 individuals per day, indicating that approximately 1% of the population is treated with the respective drug on a daily basis [13, 15]. One key advantage of DID is that it serves as a standardized, internationally comparable measure of treatment intensity over time, independent of the size of the population, pack sizes, substance strength, or formulation [16].

It should be noted that the DDDs used in this analysis were not entirely based on the official WHO or German versions of the ATC/DDD Index. Instead, DDDs were assigned according to the prescribed dose: prasugrel 5 mg (DDD 5 mg instead of WHO 10 mg), rivaroxaban 2.5 mg (DDD 5 mg instead of WHO 20 mg), ticagrelor 60 mg (DDD 120 mg instead of WHO 180 mg), and colchicine 0.5 mg (DDD 0.5 mg instead of WHO 1.0 mg), as shown in Table 1. These DDDs were defined based on the doses given in the studies investigating long-term antithrombotic [5–7] and anti-inflammatory [3, 4] drugs in patients with CAD. For all other medications, WHO-defined DDDs were used. Utilization volumes were aggregated on a yearly basis, and additionally by calendar quarter for the year 2024. Stratified analyses were performed by prescribing physician’s specialty and by 5-year age groups of patients. All figures are created with RStudio (Posit PBC, Boston, MA, USA).

### Definition of physician specialties

The DAPI database enables identification of the prescribing physician’s specialty based on physician group codes provided by the National Association of Statutory Health Insurance Physicians (Kassenärztliche Bundesvereinigung). Using these codes, each prescription was assigned to a specific medical specialty. For the purpose of this analysis, prescriptions were assigned to the following physician specialties: general practitioners, cardiologists, internists, angiologists, orthopedic specialists, gastroenterologists, rheumatologists, and gynaecologists. Gastroenterologists and gynaecologists were included as control groups, as these specialties rarely prescribe cardiovascular medications or colchicine for non-cardiovascular indications such as gout.

## Results

**Fig. 1:**
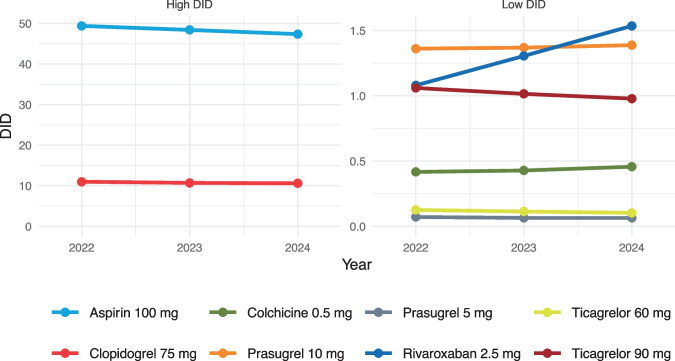
Defined daily doses per 1000 SH-insured persons per day (DID) 2022–2024. Left side: Aspirin and clopidogrel (high DID). Right side: Colchicine, prasugrel, rivaroxaban, and ticagrelor (low DID). SH, Statutory Health (Insurance funds)

From 2022 to 2024, dispensing volumes of aspirin, clopidogrel, colchicine, prasugrel (5 mg and 10 mg), and ticagrelor (60 mg and 90 mg) remained largely stable (Table 2 and Fig. 1). The use of rivaroxaban 2.5 mg increased by 42%, rising from 1.08 DID in 2022 to 1.535 DID in 2024 (Fig. 1). Among all substances studied, aspirin accounted for the highest dispensing volume in 2024 (47.377 DID), corresponding to approximately 4.7% of the German population aged > 45 receiving aspirin daily. Clopidogrel had the second highest and stable utilization (10.625 DID in 2024). The remaining substances, colchicine, prasugrel (5 mg and 10 mg), rivaroxaban (2.5 mg), and ticagrelor (60 mg and 90 mg), were prescribed considerably less frequently (Table 2). Notably, low-dose prasugrel (5 mg) was dispensed far less often than the standard 10 mg dose (0.065 vs. 1.388 DID in 2024), and ticagrelor 60 mg less than ticagrelor 90 mg (0.103 vs. 0.978 DID in 2024). Colchicine, used as a low-dose anti-inflammatory agent, was also rarely used (0.457 DID in 2024).

**Table 2:** Dispensed defined daily doses per 1000 SH-insured persons per day (DID) 2022–2024

Drug	2022		2023		2024	
	Total	Percent	Total	Percent	Total	Percent
Aspirin 100 mg	49.395	76.58%	48.421	76.34%	47.377	75.77%
Clopidogrel 75 mg	10.988	17.04%	10.707	16.88%	10.625	16.99%
Colchicine 0.5 mg	0.417	0.65%	0.428	0.67%	0.457	0.73%
Prasugrel 10 mg	1.361	2.11%	1.369	2.16%	1.388	2.22%
Prasugrel 5 mg	0.072	0.11%	0.065	0.10%	0.065	0.10%
*Rivaroxaban 2.5 mg*	*1.08*	*1.67%*	*1.305*	*2.06%*	*1.535*	*2.45%*
Ticagrelor 60 mg	0.126	0.20%	0.114	0.18%	0.103	0.16%
Ticagrelor 90 mg	1.06	1.64%	1.015	1.60%	0.978	1.56%

*SH* Statutory Health (Insurance funds)

Marked in italics: Dispensings of rivaroxaban 2.5 mg increased by 42% from 2022 to 2024

**Fig. 2:**
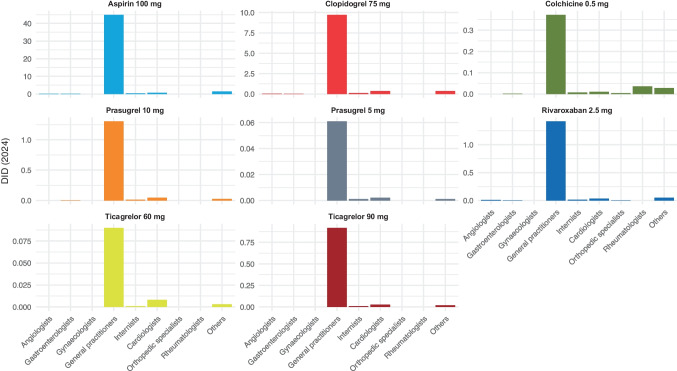
Defined daily doses per 1000 SH-insured persons per day (DID) by prescribing physician specialty (2024). SH, Statutory Health (Insurance funds)

Across all investigated substances, general practitioners were the predominant prescribers (Fig. 2 and online supplemental Table S1). Cardiologists accounted for a small share of total prescriptions, with a mean DID contribution of 3.3% and 1.8% of all dispensed DDDs. In 2024, cardiologist prescription share was: 1.3% for aspirin, 3.5% for clopidogrel, 2.2% for colchicine 0.5 mg, 3.1% for prasugrel 10 mg, 3.1% for prasugrel 5 mg, 2.2% for rivaroxaban 2.5 mg, 7.8% for ticagrelor 60 mg, and 2.9% for ticagrelor 90 mg. For colchicine, general practitioners issued 81.2% of all prescriptions (0.37 DID), followed by rheumatologists at 7.9% of total prescriptions (0.036 DID).

**Fig. 3:**
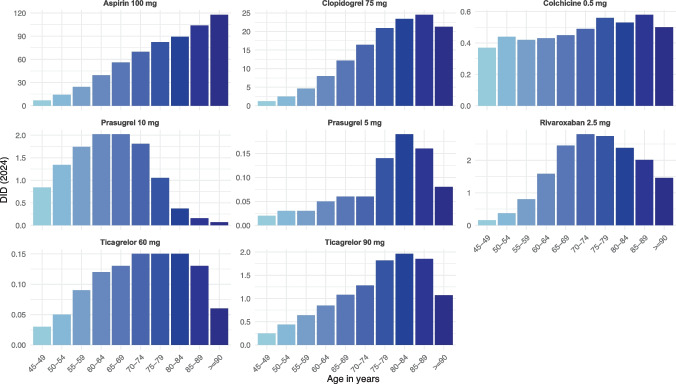
Defined daily doses per 1000 SH-insured persons per day (DID) by patients’ age group (2024). SH, Statutory Health (Insurance funds)

A continuous increase in aspirin use with age was observed (Fig. 3). In 2024, aspirin DID rose from 6.86 in individuals aged 45–49 years to 117.79 in those aged > 90 years, indicating that approximately 11.8% of the ≥ 90-year-old population received aspirin daily (online supplemental Table S2). Similarly, clopidogrel dispensing followed a similar age-related pattern, peaking in the 85–89 years’ age group in 2024, and slightly declined in individuals aged ≥ 90 years. Colchicine prescriptions remained consistently low across age groups, with minimal variation. Prasugrel 10 mg use peaked in patients aged 60–69 years, followed by a marked decline in older age groups. Prasugrel 5 mg, in turn, was rarely prescribed in younger patients and showed a slight trend toward increased use in older individuals. Rivaroxaban 2.5 mg peaked in the 70–74 years’ age group and gradually declined thereafter. Ticagrelor 60 mg was most frequently dispensed to patients aged 70–84 years, while ticagrelor 90 mg peaked in a slightly older population.

**Fig. 4:**
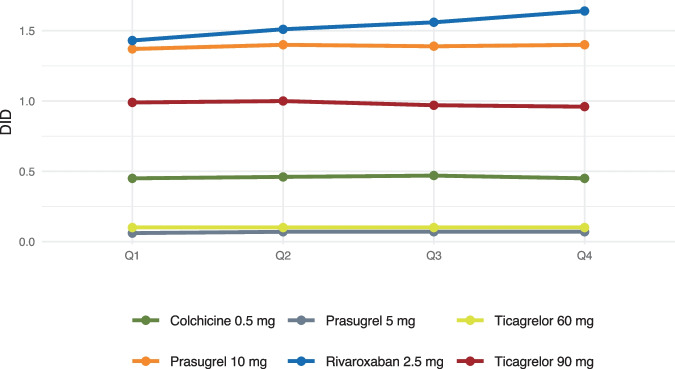
Defined daily doses per 1000 SH-insured persons per day (DID) by quarter (Q) 1–4 2024 (aspirin and clopidogrel excluded). SH, Statutory Health (Insurance funds)

Following the availability of generic formulations of rivaroxaban 2.5 mg in early 2024 [17], quarterly analysis revealed a continued increase in dispensing volume throughout the year (Fig. 4). The DID rose from 1.43 in Q1 to 1.64 in Q4, corresponding to a 14.7% relative increase (online supplemental Table S3). However, a DID of 1.64 indicates that approximately only 0.164% of the population received this drug daily. Consequently, in absolute terms, the prescription rates were low despite the high prevalence of CAD in Germany. In contrast, quarterly utilization of all other included substances remained largely unchanged.

## Discussion

In this analysis of German outpatient prescription data from 2022 to 2024, the use of guideline-recommended long-term antithrombotic and anti-inflammatory therapies was investigated. While most substances showed stable dispensing volumes over time, rivaroxaban 2.5 mg demonstrated a notable 42% increase, likely due to the expiration of patent protection and availability of low-cost generics [17].

Despite strong evidence from large clinical trials and consistent guideline recommendations, the overall use of low-dose long-term therapies remained low, with aspirin by far the most frequently prescribed agent. Prescription rates for rivaroxaban 2.5 mg, ticagrelor 60 mg, prasugrel 5 mg, and colchicine were substantially lower, with general practitioners responsible for the majority of prescriptions across all investigated medications.

CAD continues to be a major contributor to global morbidity and mortality [18, 19]. In 2019, ischemic heart disease was responsible for over 197 million prevalent cases, 9.1 million deaths, and 182 million disability-adjusted life years worldwide [19]. A considerable proportion of patients with established CAD continue to face a high residual ischemic risk [1, 20]. In a registry study including 97,254 post-MI patients, 18.3% experienced a recurrent cardiovascular event (defined as non-fatal MI, non-fatal stroke, or cardiovascular death) within the first 365 days following hospital discharge [20]. The risk remained high even beyond the first year, with 20% of initially stable patients experiencing such events during the following 36 months, underscoring the need for sustained secondary prevention [20].

The German DEGS1 study provided representative data on the prevalence of CAD in Germany among adults aged 40–79 years [21]. It showed an overall lifetime prevalence of 9.3%, with an age and sex gradient: 12.3% in men and 6.4% in women, rising from 2.3% in those aged 40–49 years to 22.3% in those aged 70–79 years [21]. Given this high prevalence, the observed prescription rates for evidence-based therapies in our study appear disproportionately low. For instance, the DID of low-dose rivaroxaban in Q4 2024 was 1.64 and peaked in patients aged 70–74 years, indicating that approximately only 0.164% of the population received this drug daily. Two large real-world analyses have applied the COMPASS eligibility criteria to unselected patient populations. These studies indicated that a substantial proportion (37–45%) of contemporary CAD/ASCVD patients would meet the COMPASS eligibility criteria [22, 23], supporting the view that a relevant share of our target population could be candidates for long-term dual antithrombotic therapy. Furthermore, a substantial proportion of post-MI patients would also meet the PEGASUS-TIMI 54 eligibility criteria, with two large observational studies reporting eligibility rates ranging from 41.1% to 77.5% [24, 25]. Consequently, our data suggest an underuse of long-term strategies in clinical care.

Several trials have demonstrated the benefit of extended antithrombotic therapy [5–8]. The number needed to treat (NNT) to prevent one ischemic event was 63 for clopidogrel and prasugrel (DAPT), 77 for rivaroxaban 2.5 mg (COMPASS), and 84 for ticagrelor (PEGASUS), with corresponding numbers needed to harm (NNH) for major bleeding of 105 for clopidogrel and prasugrel, 84 for rivaroxaban, and 81 for ticagrelor, respectively [11]. The 2020 and 2023 ESC Guidelines for the management of ACS recommend the addition of a second antithrombotic agent—such as low-dose rivaroxaban, ticagrelor 60 mg, clopidogrel, or prasugrel—to aspirin for extended long-term secondary prevention in selected high ischemic risk patients without high bleeding risk (Class IIa, A recommendation) [10, 11]. Despite this endorsement in 2020, the renewed recommendation in 2023, and the high number of patients with high ischemic risk, the findings of this study show low implementation of extended antithrombotic strategies in Germany. Even 1 year after the second guideline release, in 2024, dispensing volumes of these therapies remained low across all substance groups, suggesting guideline inertia.

Rivaroxaban 2.5 mg was the only therapy with increased use, likely due to substantial cost reduction for generics following patent expiry [17]. In contrast, low-dose prasugrel 5 mg and ticagrelor 60 mg, both recommended for long-term secondary prevention, remained rarely prescribed. In addition to cost-related factors, the higher prescription rates of rivaroxaban 2.5 mg compared with prasugrel 5 mg or ticagrelor 60 mg may also reflect its broader spectrum of approved indications. Unlike prasugrel 5 mg or ticagrelor 60 mg, which are indicated only in patients after ACS [5, 7, 9], rivaroxaban 2.5 mg is also approved for long-term secondary prevention in patients with CCS and in patients with peripheral artery disease (PAD) [6]. This wider indication spectrum likely expands the pool of eligible patients. However, as our dataset does not provide information on the specific indication underlying each prescription, this explanation remains speculative. In addition, the demographic shift with an aging population and the projected rise in the prevalence of cardiovascular disease could further contribute to the growing pool of patients eligible for low-dose rivaroxaban therapy [26]. Even for agents more commonly used (e.g., clopidogrel 75 mg, prasugrel 10 mg, ticagrelor 90 mg), use likely reflects early post-ACS management rather than long-term secondary prevention, though this cannot be verified from dispensing data alone. Notably, utilization declined in patients aged > 80 years, despite their elevated residual ischemic risk. Cardiologists did not show significantly higher prescribing rates than general practitioners, suggesting therapeutic inertia. Similarly, angiologists seem to prescribe rivaroxaban infrequently in patients with PAD. Overall, these findings indicate a significant underuse of prolonged antithrombotic therapy in routine clinical practice.

Several factors may contribute to the underuse of prolonged antithrombotic therapy, such as concerns over bleeding, particularly in elderly patients, and a lack of awareness among clinicians regarding the efficacy of these therapies and their indication in patients with high residual ischemic risk. Another possible reason is the perception that elderly patients may derive limited benefit from long-term therapy, despite their elevated baseline risk. Although all investigated agents (low-dose rivaroxaban, ticagrelor, and prasugrel in combination with aspirin) were associated with higher rates of major bleeding compared to aspirin monotherapy, the absolute rates remained low. Importantly, none of these agents increased the risk of fatal bleeding or intracranial hemorrhage compared to aspirin monotherapy [5–7]. These findings align with the previously discussed NNT and NNH values, which illustrate the balance between ischemic event prevention and bleeding risk. Of note, prasugrel 5 mg is not formally approved in Germany for extended secondary prevention beyond 12 months. According to the current prescribing information of prasugrel (Efient®), “a treatment duration of up to 12 months is recommended.” While this wording does not explicitly prohibit longer use, it reflects the evidence base available at the time of approval. As such, prasugrel 5 mg is considered off-label when used for prolonged maintenance therapy, which likely influences real-world prescribing behavior. In particular, the lack of a formal label and the associated lack of reimbursement in the German outpatient health care system may defer physicians from prescribing prasugrel 5 mg for long-term secondary prevention, despite supportive clinical trial evidence. A careful patient selection and individualized risk–benefit assessment, particularly weighing ischemic versus hemorrhagic risk, are necessary. Nonetheless, median ages in pivotal trials ranged from 60 to 70 years, leaving limited evidence for the very elderly. In such cases, clopidogrel may offer a safer option due to its lower bleeding risk [27, 28].

According to the 2024 ESC Guidelines for the management of chronic coronary syndromes, low-dose colchicine should be considered in patients with atherosclerotic CAD to reduce MI, stroke, and the need for revascularization (Class IIa, A) [12]. However, in Germany, colchicine is not approved for cardiovascular prevention but for gout, prevention of amyloidosis, and familial Mediterranean fever [29]. Herein, liquid formulations of colchicine were excluded based on the assumption that liquid colchicine is primarily prescribed for pediatric indications and not for CAD in adults. Prescribing was dominated by general practitioners and rheumatologists, most likely for gout rather than for secondary prevention after MI, with cardiologists accounting for a small fraction. It is also likely that a not quantifiable proportion of colchicine prescriptions by cardiologists were related to the treatment of pericarditis, although not approved for this indication, rather than ASCVD, which may further overestimate its use for event prevention in patients with CAD. Although colchicine is an inexpensive drug (net cost per 0.5 mg EUR 0.82 in 2023) with a favorable efficacy profile at low dose, its use in the context of ASCVD remains limited: In this analysis, cardiologists prescribed 3679 packs (30 tablets each) in 2022 and 4390 packs in 2024. Unlike in the USA, where the Food and Drug Administration (FDA) has approved low-dose colchicine for the prevention of recurrent cardiovascular events [30], it is currently not approved by the European Medicines Agency (EMA) for this indication [31]. Possible reasons for the limited use in Germany include restricted awareness and concerns over off-label prescribing, possible drug-drug interactions, liability, and tolerability [31]. A recent modeling study estimated that widespread use of colchicine for secondary prevention in eligible US adults could prevent approximately 226,000 major adverse cardiovascular events over 3 years, yet real-world uptake remains low, mirroring our findings from Germany [32]. However, the CLEAR trial investigated the use of colchicine (0.5 mg daily) in 7062 patients following acute MI and found no significant reduction in the composite cardiovascular endpoint over a median follow-up of 3 years [33]. These results contrast with previous trials COLCOT and LoDoCo2, which had demonstrated a significant benefit of colchicine in both post-MI and stable CAD populations [3, 4].

Our findings reflect the outpatient prescription landscape within the SH-insurance system in Germany, which covers approximately 87% of the population and is subject to reimbursement regulations and potential cost reclaims. While such financial constraints may impact prescribing behavior, cost considerations should not compromise evidence-based care or restrict access to therapies with proven benefit. Economic evaluations have shown that rivaroxaban plus aspirin is cost-effective in the USA [34], while ticagrelor 60 mg plus aspirin provided intermediate value overall but became economically favorable in high-risk patient groups [35]. These findings underscore that the targeted use of such therapies is both clinically and economically justified.

To the best of our knowledge, no comparable study has systematically evaluated nationwide prescription data for long-term antithrombotic therapies in patients with CAD. Our analysis is the first to provide national-level data demonstrating that the prescription of these evidence-based therapies is rare. The very low dispensing volumes strongly suggest that these therapies are only rarely implemented in clinical routine. To date, there is only one published analysis of colchicine prescribing in the context of cardiovascular prevention: a brief report from the USA using IQVIA’s national prescription audit data from 2018 to 2024 [36]. In this study, colchicine prescription trends were quantified, and the authors showed that cardiologists accounted for only 2.8–4.0% of monthly prescriptions nationwide, despite increasing evidence and FDA approval for CAD [36]. Although monthly prescriptions rose slightly after the LoDoCo2 trial [3] and subsequent approval of colchicine, the overall uptake remained modest, with an estimated 4000 additional prescriptions per month attributable to cardiovascular indications [36]. These findings align with our results from Germany. However, our study extends previous data in several ways: (1) we provide detailed stratification by age and prescribing specialty, (2) we report DID to allow population-level estimates of drug exposure, and (3) our analysis also includes data for antithrombotic therapies, providing a more comprehensive picture of real-world guideline implementation within this context.

### Strengths and limitations

Strengths of this study include the use of a comprehensive nationwide database, covering over 95% of community pharmacies and approximately 87% of the German population. The 3-year observation period captures temporal trends around two successive ESC guideline updates. Stratification by age and prescriber specialty enabled detailed subgroup analyses and indirect inference regarding treatment intent.

Limitations include the lack of clinical diagnoses, comorbidities, or indications, limiting direct linkage between prescriptions and cardiovascular events or patient characteristics. Therefore, the distinction between short-term and long-term use (e.g., within 12 months post-ACS) was not possible. Hospital-dispensed medications and those covered by private insurance were not captured, potentially underestimating total drug use by approximately 10%. Adherence or persistence data were unavailable, and DID metrics may not reflect individualized dosing or patient-level variation in treatment duration and intensity. Lastly, results are specific to Germany and may not be generalizable to other healthcare systems or countries.

## Conclusion

This study provides the first national-level assessment of outpatient prescription patterns for long-term antithrombotic and anti-inflammatory therapies in Germany. Despite strong evidence and repeated guideline recommendations, the implementation of long-term low-dose antithrombotic and anti-inflammatory therapies in patients with CAD remains limited, particularly among older patients. These findings highlight a substantial gap between guideline-directed care and real-world prescribing, emphasizing the need for improved awareness, education, and implementation strategies to optimize secondary prevention in patients at risk.

## Supplementary Information

Below is the link to the electronic supplementary material.

Supplementary file1 (DOCX 48 KB)

Supplementary file2 (DOCX 45 KB)

Supplementary file3 (DOCX 32 KB)

## Data Availability

The data underlying this article cannot be shared publicly due to restricted access to the DAPI database and was granted for the current analysis. The data are not publicly available due to data protection regulations but will be shared on reasonable request to the corresponding author with permission from the DAPI.
