# Supplementary material for: Temporal trends in the prescription of low-dose antithrombotic and anti-inflammatory therapies in Germany (2022–2024)
Source: Clin Res Cardiol. 2025 Oct 7;115(8):1351–61. doi: 10.1007/s00392-025-02761-x (PMC13346128; doi:10.1007/s00392-025-02761-x)
Supplement: Supplementary file 1 — Supplementary file1 (DOCX 48 KB) [file 392_2025_2761_MOESM1_ESM.docx]

**Supplemental Table S1 Defined Daily Doses per 1,000 SH-insured persons per day (DID) by prescribing physician specialty (2022 – 2024)**

|  | **2022** | **2022** | **2023** | **2023** | **2024** | **2024** |
| --- | --- | --- | --- | --- | --- | --- |
|  | **Total DID** | **Percent DID** | **Total DID** | **Percent DID** | **Total DID** | **Percent DID** |
| **Aspirin 100 mg** | **49.396** | **100%** | **48.421** | **100%** | **47.378** | **100%** |
| Angiologists | 0.035 | 0.07% | 0.034 | 0.07% | 0.028 | 0.06% |
| Gastroenterologists | 0.041 | 0.08% | 0.037 | 0.08% | 0.032 | 0.07% |
| Gynaecologists | 0.004 | 0.01% | 0.004 | 0.01% | 0.004 | 0.01% |
| General practitioners | 46.766 | 94.68% | 45.781 | 94.55% | 44.906 | 94.78% |
| Internists | 0.461 | 0.93% | 0.432 | 0.89% | 0.404 | 0.85% |
| Cardiologists | 0.619 | 1.25% | 0.65 | 1.34% | 0.633 | 1.34% |
| Orthopedic specialists | 0.012 | 0.02% | 0.012 | 0.02% | 0.013 | 0.03% |
| Rheumatologists | 0.016 | 0.03% | 0.015 | 0.03% | 0.014 | 0.03% |
| Others | 1.442 | 2.92% | 1.456 | 3.01% | 1.344 | 2.84% |
| **Clopidogrel 75 mg** | **10.987** | **100%** | **10.706** | **100%** | **10.626** | **100%** |
| Angiologists | 0.028 | 0.25% | 0.026 | 0.24% | 0.024 | 0.23% |
| Gastroenterologists | 0.01 | 0.09% | 0.009 | 0.08% | 0.009 | 0.08% |
| Gynaecologists | 0.001 | 0.01% | 0.001 | 0.01% | 0.001 | 0.01% |
| General practitioners | 10.062 | 91.58% | 9.788 | 91.43% | 9.727 | 91.54% |
| Internists | 0.141 | 1.28% | 0.134 | 1.25% | 0.124 | 1.17% |
| Cardiologists | 0.353 | 3.21% | 0.359 | 3.35% | 0.372 | 3.50% |
| Orthopedic specialists | 0.002 | 0.02% | 0.002 | 0.02% | 0.002 | 0.02% |
| Rheumatologists | 0.003 | 0.03% | 0.003 | 0.03% | 0.003 | 0.03% |
| Others | 0.387 | 3.52% | 0.384 | 3.59% | 0.364 | 3.43% |
| **Colchicine 0.5 mg** | **0.417** | **100%** | **0.428** | **100%** | **0.458** | **100%** |
| Angiologists | 0 | 0.00% | 0 | 0.00% | 0 | 0.00% |
| Gastroenterologists | 0.001 | 0.24% | 0.001 | 0.23% | 0.001 | 0.22% |
| Gynaecologists | 0 | 0.00% | 0 | 0.00% | 0 | 0.00% |
| General practitioners | 0.347 | 83.21% | 0.353 | 82.48% | 0.372 | 81.22% |
| Internists | 0.006 | 1.44% | 0.007 | 1.64% | 0.007 | 1.53% |
| Cardiologists | 0.008 | 1.92% | 0.008 | 1.87% | 0.01 | 2.18% |
| Orthopedic specialists | 0.005 | 1.20% | 0.004 | 0.93% | 0.004 | 0.87% |
| Rheumatologists | 0.025 | 6.00% | 0.029 | 6.78% | 0.036 | 7.86% |
| Others | 0.025 | 6.00% | 0.026 | 6.07% | 0.028 | 6.11% |
| **Prasugrel 10 mg** | **1.36** | **100%** | **1.369** | **100%** | **1.387** | **100%** |
| Angiologists | 0.001 | 0.07% | 0.001 | 0.07% | 0 | 0.00% |
| Gastroenterologists | 0.001 | 0.07% | 0.001 | 0.07% | 0.001 | 0.07% |
| Gynaecologists | 0 | 0.00% | 0 | 0.00% | 0 | 0.00% |
| General practitioners | 1.272 | 93.53% | 1.282 | 93.64% | 1.305 | 94.09% |
| Internists | 0.015 | 1.10% | 0.015 | 1.10% | 0.014 | 1.01% |
| Cardiologists | 0.046 | 3.38% | 0.045 | 3.29% | 0.043 | 3.10% |
| Orthopedic specialists | 0 | 0.00% | 0 | 0.00% | 0 | 0.00% |
| Rheumatologists | 0 | 0.00% | 0 | 0.00% | 0 | 0.00% |
| Others | 0.025 | 1.84% | 0.025 | 1.83% | 0.024 | 1.73% |
| **Prasugrel 5 mg** | **0.072** | **100%** | **0.064** | **100%** | **0.065** | **100%** |
| Angiologists | 0 | 0.00% | 0 | 0.00% | 0 | 0.00% |
| Gastroenterologists | 0 | 0.00% | 0 | 0.00% | 0 | 0.00% |
| Gynaecologists | 0 | 0.00% |  |  | 0 | 0.00% |
| General practitioners | 0.066 | 91.67% | 0.06 | 93.75% | 0.061 | 93.85% |
| Internists | 0.001 | 1.39% | 0.001 | 1.56% | 0.001 | 1.54% |
| Cardiologists | 0.003 | 4.17% | 0.002 | 3.13% | 0.002 | 3.08% |
| Orthopedic specialists | 0 | 0.00% |  |  | 0 | 0.00% |
| Rheumatologists | 0 | 0.00% | 0 | 0.00% | 0 | 0.00% |
| Others | 0.002 | 2.78% | 0.001 | 1.56% | 0.001 | 1.54% |
| **Rivaroxaban 2.5 mg** | **1.081** | **100%** | **1.306** | **100%** | **1.534** | **100%** |
| Angiologists | 0.011 | 1.02% | 0.012 | 0.92% | 0.012 | 0.78% |
| Gastroenterologists | 0.001 | 0.09% | 0.001 | 0.08% | 0.001 | 0.07% |
| Gynaecologists | 0 | 0.00% | 0 | 0.00% | 0 | 0.00% |
| General practitioners | 0.979 | 90.56% | 1.195 | 91.50% | 1.421 | 92.63% |
| Internists | 0.014 | 1.30% | 0.015 | 1.15% | 0.014 | 0.91% |
| Cardiologists | 0.031 | 2.87% | 0.033 | 2.53% | 0.034 | 2.22% |
| Orthopedic specialists | 0.001 | 0.09% | 0.001 | 0.08% | 0.001 | 0.07% |
| Rheumatologists | 0 | 0.00% | 0 | 0.00% | 0 | 0.00% |
| Others | 0.044 | 4.07% | 0.049 | 3.75% | 0.051 | 3.32% |
| **Ticagrelor 60 mg** | **0.125** | **100%** | **0.114** | **100%** | **0.102** | **100%** |
| Angiologists | 0 | 0.00% | 0 | 0.00% | 0 | 0.00% |
| Gastroenterologists | 0 | 0.00% | 0 | 0.00% | 0 | 0.00% |
| Gyneacologists | 0 | 0.00% | 0 | 0.00% |  |  |
| General practitioners | 0.111 | 88.80% | 0.101 | 88.60% | 0.09 | 88.24% |
| Internists | 0.002 | 1.60% | 0.002 | 1.75% | 0.001 | 0.98% |
| Cardiologists | 0.009 | 7.20% | 0.008 | 7.02% | 0.008 | 7.84% |
| Orthopedic specialists | 0 | 0.00% | 0 | 0.00% | 0 | 0.00% |
| Rheumatologists | 0 | 0.00% | 0 | 0.00% |  |  |
| Others | 0.003 | 2.40% | 0.003 | 2.63% | 0.003 | 2.94% |
| **Ticagrelor 90 mg** | **1.06** | **100%** | **1.015** | **100%** | **0.977** | **100%** |
| Angiologists | 0.001 | 0.09% | 0.001 | 0.10% | 0.001 | 0.10% |
| Gastroenterologists | 0.001 | 0.09% | 0.001 | 0.10% | 0.001 | 0.10% |
| Gynaecologists | 0 | 0.00% | 0 | 0.00% | 0 | 0.00% |
| General practitioners | 0.994 | 93.77% | 0.951 | 93.69% | 0.919 | 94.06% |
| Internists | 0.012 | 1.13% | 0.011 | 1.08% | 0.009 | 0.92% |
| Cardiologists | 0.03 | 2.83% | 0.029 | 2.86% | 0.028 | 2.87% |
| Orthopedic specialists | 0 | 0.00% | 0 | 0.00% | 0 | 0.00% |
| Rheumatologists | 0 | 0.00% | 0 | 0.00% | 0 | 0.00% |
| Others | 0.022 | 2.08% | 0.022 | 2.17% | 0.019 | 1.94% |

SH = Statutory Health (Insurance funds)
