# Supplementary material for: Temporal trends in the prescription of low-dose antithrombotic and anti-inflammatory therapies in Germany (2022–2024)
Source: Clin Res Cardiol. 2025 Oct 7;115(8):1351–61. doi: 10.1007/s00392-025-02761-x (PMC13346128; doi:10.1007/s00392-025-02761-x)
Supplement: Supplementary file 2 — Supplementary file2 (DOCX 45 KB) [file 392_2025_2761_MOESM2_ESM.docx]

**Supplemental Table S2 Defined Daily Doses per 1,000 SH-insured persons per day (DID) by patients’ age group (2022 – 2024)**

| **Age** | **Total DID** | | | **Percent DID** |
| --- | --- | --- | --- | --- |
| **Drug** | **2022** | **2023** | **2024** | **2024** |
| **45 – 49 years** | **10.43** | **10.13** | **9.72** | **100.0%** |
| Aspirin 100 mg | 7.49 | 7.22 | 6.86 | 70.6% |
| Clopidogrel 75 mg | 1.25 | 1.23 | 1.20 | 12.4% |
| Colchicine 0.5 mg | 0.35 | 0.34 | 0.37 | 3.8% |
| Prasugrel 10 mg | 0.86 | 0.86 | 0.84 | 8.6% |
| Prasugrel 5 mg | 0.02 | 0.02 | 0.02 | 0.2% |
| Rivaroxaban 2.5 mg | 0.14 | 0.15 | 0.16 | 1.7% |
| Ticagrelor 60 mg | 0.04 | 0.04 | 0.03 | 0.3% |
| Ticagrelor 90 mg | 0.30 | 0.27 | 0.25 | 2.5% |
| **50 – 54 years** | **20.50** | **20.12** | **19.29** | **100.0%** |
| Aspirin 100 mg | 15.23 | 14.89 | 14.14 | 73.3% |
| Clopidogrel 75 mg | 2.62 | 2.57 | 2.49 | 12.9% |
| Colchicine 0.5 mg | 0.37 | 0.39 | 0.44 | 2.3% |
| Prasugrel 10 mg | 1.34 | 1.35 | 1.34 | 6.9% |
| Prasugrel 5 mg | 0.03 | 0.03 | 0.03 | 0.1% |
| Rivaroxaban 2.5 mg | 0.31 | 0.34 | 0.37 | 1.9% |
| Ticagrelor 60 mg | 0.08 | 0.07 | 0.05 | 0.3% |
| Ticagrelor 90 mg | 0.53 | 0.49 | 0.44 | 2.3% |
| **55 – 59 years** | **34.63** | **33.64** | **32.73** | **100.0%** |
| Aspirin 100 mg | 26.11 | 25.31 | 24.40 | 74.5% |
| Clopidogrel 75 mg | 4.91 | 4.71 | 4.63 | 14.1% |
| Colchicine 0.5 mg | 0.37 | 0.38 | 0.42 | 1.3% |
| Prasugrel 10 mg | 1.72 | 1.71 | 1.74 | 5.3% |
| Prasugrel 5 mg | 0.04 | 0.03 | 0.03 | 0.1% |
| Rivaroxaban 2.5 mg | 0.65 | 0.73 | 0.80 | 2.4% |
| Ticagrelor 60 mg | 0.11 | 0.10 | 0.09 | 0.3% |
| Ticagrelor 90 mg | 0.73 | 0.67 | 0.64 | 2.0% |
| **60 – 64 years** | **55.32** | **53.92** | **52.58** | **100.0%** |
| Aspirin 100 mg | 42.04 | 40.83 | 39.52 | 75.2% |
| Clopidogrel 75 mg | 8.51 | 8.19 | 7.99 | 15.2% |
| Colchicine 0.5 mg | 0.38 | 0.41 | 0.43 | 0.8% |
| Prasugrel 10 mg | 1.97 | 1.96 | 2.02 | 3.8% |
| Prasugrel 5 mg | 0.05 | 0.05 | 0.05 | 0.1% |
| Rivaroxaban 2.5 mg | 1.22 | 1.42 | 1.59 | 3.0% |
| Ticagrelor 60 mg | 0.15 | 0.13 | 0.12 | 0.2% |
| Ticagrelor 90 mg | 1.00 | 0.93 | 0.85 | 1.6% |
| **65 – 69 years** | **76.70** | **75.82** | **74.51** | **100.0%** |
| Aspirin 100 mg | 58.55 | 57.63 | 56.15 | 75.4% |
| Clopidogrel 75 mg | 12.68 | 12.35 | 12.17 | 16.3% |
| Colchicine 0.5 mg | 0.40 | 0.42 | 0.45 | 0.6% |
| Prasugrel 10 mg | 1.96 | 1.98 | 2.02 | 2.7% |
| Prasugrel 5 mg | 0.05 | 0.05 | 0.06 | 0.1% |
| Rivaroxaban 2.5 mg | 1.72 | 2.10 | 2.45 | 3.3% |
| Ticagrelor 60 mg | 0.16 | 0.15 | 0.13 | 0.2% |
| Ticagrelor 90 mg | 1.17 | 1.14 | 1.08 | 1.4% |
| **70 – 74 years** | **94.44** | **93.53** | **92.90** | **100.0%** |
| Aspirin 100 mg | 71.87 | 70.92 | 69.90 | 75.2% |
| Clopidogrel 75 mg | 16.91 | 16.52 | 16.42 | 17.7% |
| Colchicine 0.5 mg | 0.47 | 0.47 | 0.49 | 0.5% |
| Prasugrel 10 mg | 1.74 | 1.76 | 1.81 | 1.9% |
| Prasugrel 5 mg | 0.06 | 0.06 | 0.06 | 0.1% |
| Rivaroxaban 2.5 mg | 1.87 | 2.33 | 2.80 | 3.0% |
| Ticagrelor 60 mg | 0.17 | 0.16 | 0.15 | 0.2% |
| Ticagrelor 90 mg | 1.36 | 1.31 | 1.28 | 1.4% |
| **75 – 79 years** | **111.30** | **110.46** | **109.67** | **100.0%** |
| Aspirin 100 mg | 84.38 | 83.47 | 82.34 | 75.1% |
| Clopidogrel 75 mg | 21.46 | 21.11 | 20.89 | 19.0% |
| Colchicine 0.5 mg | 0.51 | 0.53 | 0.56 | 0.5% |
| Prasugrel 10 mg | 0.95 | 1.01 | 1.05 | 1.0% |
| Prasugrel 5 mg | 0.15 | 0.14 | 0.14 | 0.1% |
| Rivaroxaban 2.5 mg | 1.85 | 2.26 | 2.74 | 2.5% |
| Ticagrelor 60 mg | 0.18 | 0.16 | 0.15 | 0.1% |
| Ticagrelor 90 mg | 1.82 | 1.79 | 1.82 | 1.7% |
| **80 – 84 years** | **125.05** | **121.21** | **118.31** | **100.0%** |
| Aspirin 100 mg | 95.64 | 92.38 | 89.33 | 75.5% |
| Clopidogrel 75 mg | 24.39 | 23.58 | 23.42 | 19.8% |
| Colchicine 0.5 mg | 0.52 | 0.52 | 0.53 | 0.5% |
| Prasugrel 10 mg | 0.41 | 0.39 | 0.37 | 0.3% |
| Prasugrel 5 mg | 0.23 | 0.20 | 0.19 | 0.2% |
| Rivaroxaban 2.5 mg | 1.64 | 2.00 | 2.38 | 2.0% |
| Ticagrelor 60 mg | 0.17 | 0.15 | 0.15 | 0.1% |
| Ticagrelor 90 mg | 2.06 | 2.00 | 1.96 | 1.7% |
| **85 -89 years** | **141.89** | **137.26** | **133.35** | **100.0%** |
| Aspirin 100 mg | 111.69 | 107.82 | 103.95 | 78.0% |
| Clopidogrel 75 mg | 25.84 | 24.88 | 24.52 | 18.4% |
| Colchicine 0.5 mg | 0.57 | 0.57 | 0.58 | 0.4% |
| Prasugrel 10 mg | 0.20 | 0.18 | 0.16 | 0.1% |
| Prasugrel 5 mg | 0.19 | 0.17 | 0.16 | 0.1% |
| Rivaroxaban 2.5 mg | 1.34 | 1.63 | 2.01 | 1.5% |
| Ticagrelor 60 mg | 0.14 | 0.13 | 0.13 | 0.1% |
| Ticagrelor 90 mg | 1.93 | 1.87 | 1.85 | 1.4% |
| **≥ 90 years** | **148.63** | **146.80** | **142.29** | **100.0%** |
| Aspirin 100 mg | 123.81 | 122.15 | 117.79 | 82.8% |
| Clopidogrel 75 mg | 21.99 | 21.61 | 21.27 | 14.9% |
| Colchicine 0.5 mg | 0.48 | 0.47 | 0.50 | 0.4% |
| Prasugrel 10 mg | 0.06 | 0.07 | 0.07 | 0.0% |
| Prasugrel 5 mg | 0.09 | 0.07 | 0.08 | 0.1% |
| Rivaroxaban 2.5 mg | 0.97 | 1.21 | 1.46 | 1.0% |
| Ticagrelor 60 mg | 0.08 | 0.07 | 0.06 | 0.0% |
| Ticagrelor 90 mg | 1.14 | 1.13 | 1.07 | 0.8% |

SH = Statutory Health (Insurance funds)
