# Supplementary material for: Temporal trends in the prescription of low-dose antithrombotic and anti-inflammatory therapies in Germany (2022–2024)
Source: Clin Res Cardiol. 2025 Oct 7;115(8):1351–61. doi: 10.1007/s00392-025-02761-x (PMC13346128; doi:10.1007/s00392-025-02761-x)
Supplement: Supplementary file 3 — Supplementary file3 (DOCX 32 KB) [file 392_2025_2761_MOESM3_ESM.docx]

**Supplemental Table S3 Defined Daily Doses per 1,000 SH-insured persons per day (DID) by quarter (Q) 1-4 2024**

| **Quarters 2024** | **ASS**  **100 mg** | | **Clopidogrel**  **75 mg** | | **Colchicine**  **0.5 mg** | | **Prasugrel**  **10 mg** | | **Prasugrel**  **5 mg** | | **Rivaroxaban**  **2.5 mg** | | **Ticagrelor**  **60 mg** | | **Ticagrelor**  **90 mg** | |
| --- | --- | --- | --- | --- | --- | --- | --- | --- | --- | --- | --- | --- | --- | --- | --- | --- |
|  | DID | %  DID | DID | %  DID | DID | %  DID | DID | %  DID | DID | %  DID | DID | %  DID | DID | %  DID | DID | %  DID |
| **Quarter 1** | 47.39 | 76.05% | 10.53 | 16.90% | 0.45 | 0.72% | 1.37 | 2.19% | 0.06 | 0.10% | 1.43 | 2.29% | 0.10 | 0.17% | 0.99 | 1.59% |
| **Quarter 2** | 47.88 | 75.84% | 10.71 | 16.97% | 0.46 | 0.73% | 1.40 | 2.22% | 0.07 | 0.10% | 1.51 | 2.39% | 0.10 | 0.16% | 1.00 | 1.58% |
| **Quarter 3** | 46.69 | 75.50% | 10.60 | 17.15% | 0.47 | 0.75% | 1.39 | 2.24% | 0.07 | 0.11% | 1.56 | 2.52% | 0.10 | 0.16% | 0.97 | 1.56% |
| **Quarter 4** | 47.56 | 75.69% | 10.66 | 16.96% | 0.45 | 0.72% | 1.40 | 2.22% | 0.07 | 0.11% | 1.64 | 2.61% | 0.10 | 0.17% | 0.96 | 1.53% |

SH = Statutory Health (Insurance funds)
